# Pulmonary health effects of wintertime particulate matter from California and China following repeated exposure and cessation

**DOI:** 10.1016/j.toxlet.2021.10.014

**Published:** 2021-10-29

**Authors:** Wanjun Yuan, Sandra C. Velasquez, Ching-Wen Wu, Ciara C. Fulgar, Qi Zhang, Dominique E. Young, Keith J. Bein, Christoph F.A. Vogel, Wei Li, Liangliang Cui, Haiying Wei, Kent E. Pinkerton

**Affiliations:** a University of California, Davis, Center for Health and the Environment, Davis, USA; b Shanxi University, College of Environmental and Resource Sciences, Taiyuan, China; c University of California, Davis, Department of Environmental Toxicology, Davis, USA; d University of California, Davis, Air Quality Research Center, Davis, USA; e Shandong University, Biomedical Engineering Institute, School of Control Science and Engineering, Jinan, China; f Jinan Municipal Center for Disease Control and Prevention, Jinan, China

**Keywords:** PM_2.5_, Lung, Sulfate, PAHs, Time-lag, Inflammation

## Abstract

Epidemiological studies show strong associations between fine particulate matter (PM_2.5_) air pollution and adverse pulmonary effects. In the present study, wintertime PM_2.5_ samples were collected from three geographically similar regions—Sacramento, California, USA; Jinan, Shandong, China; and Taiyuan, Shanxi, China—and extracted to form PM_CA_, PM_SD_, and PM_SX_, respectively, for comparison in a BALB/c mouse model. Each of four groups was oropharyngeally administered Milli-Q water vehicle control (50 μ L) or one type of PM extract (20 μg/50 μL) five times over two weeks. Mice were necropsied on post-exposure days 1, 2, and 4 and examined using bronchoalveolar lavage (BAL), histopathology, and assessments of cytokine/chemokine mRNA and protein expression. Chemical analysis demonstrated all three extracts contained black carbon, but PM_SX_ contained more sulfates and polycyclic aromatic hydrocarbons (PAHs) associated with significantly greater neutrophil numbers and greater alveolar/bronchiolar inflammation on post-exposure days 1 and 4. On day 4, PM_SX_-exposed mice also exhibited significant increases in interleukin-1 beta, tumor necrosis factor-alpha, and chemokine C-X-C motif ligands-3 and −5 mRNA, and monocyte chemoattractant protein-1 protein. These combined findings suggest greater sulfate and PAH content contributed to a more intense and progressive inflammatory response with repeated PM_SX_ compared to PM_CA_ or PM_SD_ exposure.

## GRAPHICAL ABSTRACT

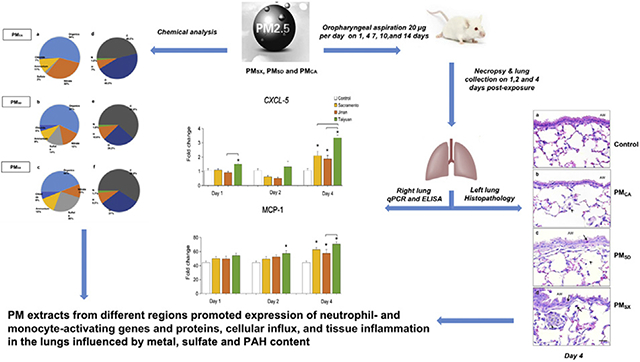

## 1. Introduction

The physicochemical characteristics of particulate matter (PM) can be highly variable due to different emission sources, weather conditions, and interactions with other solid/liquid and organic/inorganic particles suspended in the atmosphere (Kelly and Fussell, 2012). PM originating from different sources, locations, and times can exhibit unique levels of toxicity associated with respiratory effects, such as asthma and COPD (Lee et al., 2014). PM size can also greatly influence health outcomes. Although coarse PM (particles with a diameter between 10 and 2.5 μm; PM_10-2.5_) is known to irritate the eyes and nose, ambient fine PM (particles with an aerodynamic diameter <2.5 μm; PM_2.5_) has a greater potential for harmful health effects due to its ability to penetrate beyond the air passages of the upper respiratory tract and deposit in the distal lung and alveoli where gas exchange occurs (EPA, 2017; Ma et al., 2015). With clearance from the respiratory tract via mucociliary transport, lymphatic drainage, or systemic blood circulation, PM_2.5_ can also bind to cells and tissues to affect extra-pulmonary organs (Kim et al., 2015; Phalen, 2004). For these and other reasons, with more than 4.2 million premature deaths worldwide attributed to PM_2.5_ exposure in 2016 alone, PM_2.5_ is a growing universal health concern.

In the present study, PM samples were collected from cities in California (Sacramento) and China (Jinan and Taiyuan) to compare the biological effects of PM_2.5_ from different geographic locations. The cities were chosen due to their heavy urbanization, industrial and agricultural economies, moderately dry and sunny winters, and long histories of relatively high PM_2.5_ levels, especially during the winter season, when meteorological inversions are more frequent and longer in duration (Liu et al., 2010; Xia et al., 2013). California’s Central Valley has increased levels of air pollution due to its geologic bowl shape leading to the retention and accumulation of PM from vehicular exhaust, local farming, and industrial processes (Plummer et al., 2015). Several studies have shown respiratory-related emergency department visits increase with increasing PM_2.5_ concentrations (Anderson et al., 2012; Cadelis et al., 2014), and active asthma prevalence in Sacramento County individuals age 18–64 years was 12 % compared to 7.6 % for age-matched individuals state-wide. Active asthma was defined as having been diagnosed by a professional and afflicted by an asthma attack within the preceding year (CDPH, 2016).

The capital cities of Jinan and Taiyuan in the Chinese provinces of Shandong and Shanxi, respectively, represent regions of strong economic growth dominated by abundant production and industrial emissions. Shandong Province is one of the most prosperous and heavily industrialized provinces in the country, with significant coal mining, oil refineries, and developed metallurgical and mechanical sectors (Liu et al., 2018). In 2011, Jinan has experienced an annual average PM_2.5_ concentration of 149 μg/m^3^, one of the highest reported for cities worldwide (Yang et al., 2012). Similarly, Shanxi Province is China’s largest coal-producing region, with Taiyuan as one of the most polluted cities in China. From 2009 to 2010, the annual average PM_2.5_ concentration in Taiyuan was 220 μg/m^3^, more than 20 times the World Health Organization’s 10 μg/m^3^ air quality guideline for mean annual PM_2.5_ emissions (He et al., 2015; Stanway, 2017; WHO, 2018; Xia et al., 2013).

A recent study (Yuan et al., 2020) from our laboratory demonstrated a single acute exposure to extracts of PM_2.5_ collected from California, Shandong, and Shanxi (PM_CA_, PM_SD_, and PM_SX_ extracts, respectively) could cause lung inflammation and induce toxic responses in human immortalized U937 macrophages. The current study furthers our previous work by exploring pulmonary inflammatory responses to repeated oropharyngeal aspiration (OPA) of PM_CA_, PM_SD_, or PM_SX_ over two weeks, in BALB/c mice. Although comparative analyses of pulmonary inflammatory responses to PM from California and China have been reported previously (Sun et al., 2017; Yuan et al., 2020; Zhang et al., 2018), the present study is the first to assess the differential time-lag effects of repeated, low-dose exposure to PM from Sacramento, Jinan, and Taiyuan. The findings of this study are discussed in relation to previous results (Sun et al., 2017; Yuan et al., 2020; Zhang et al., 2018) to illustrate further how different exposure paradigms influence observed pulmonary responses.

## 2. Materials and methods

The methods in the present study have been reported previously (Sun et al., 2017; Yuan et al., 2020; Zhang et al., 2018) and restated herein for reader accessibility.

### 2.1. Ambient PM_2.5_ collection

Ambient winter PM_2.5_ collection occurred in Sacramento, California; Jinan, Shandong; and Taiyuan, Shanxi. The California sampling site was located in downtown Sacramento, at the northeast corner of T and 13th streets, on the rooftop of a two-story building (N38°34’, W121°29’). Downtown Jinan’s collection site was located on the rooftop of a three-story primary school, Wang She Ren (N36°40’, E117°09’). The sampling site in downtown Taiyuan was situated on the rooftop of the five-story College of Environmental Science and Resources building at Shanxi University (N37°47’, E112°34’). All three sampling locations are proximal to major freeways and surrounded by a combination of residential, commercial, and industrial sites.

In Sacramento, a high-volume sampler system (Tisch Environmental Inc., TE-6070V2.5-HVS) was used to collect PM_2.5_ for seven days. The system had a PM_2.5_ size-selective head (Tisch Environmental Inc., TE-6001) that ran at a flow rate of 40 cubic feet per minute (cfm) and was fitted with Teflon-coated borosilicate glass microfiber filters (Pall Corporation, TX40H120WW). The filters were pre-cleaned by repeated sonication in a mixture of Milli-Q water, dichloromethane, and hexane prior to sampling (Bein and Wexler, 2015).

In Jinan and Taiyuan, PM_2.5_ was collected for one day using a high-volume particle collector (Thermo Anderson, HVAIR100) operated at a flow rate of 40 cfm. The shorter collection times in China were due to higher density and heavier loading of PM relative to the sampling site in California. The sampler was fitted with a PM_2.5_ size-selective opening and 90-mm diameter quartz microfiber filters (Whatman, WHA1851090). During the 24 h before PM collection, the quartz filters were pre-heated at 450 °C for the removal of any potential endotoxin from the filters.

### 2.2. PM sample extraction

Following particle sample collection in Sacramento and Jinan, all filters were post-weighed to determine the collected PM mass (post-weight – pre-weight = PM mass). Filters were placed in Milli-Q water and sonicated for 1 h to obtain PM extracts. The sonicated PM extracts were passed through 0.2-μm pore size syringe filters, and the collected solution (approximately 100 mL) was lyophilized and stored at 80 °C. A more detailed description of the PM extraction methods was provided by Bein and Wexler(2015).

Filters from Taiyuan were post-weighed to determine the collected PM mass, subsequently cut into segments and placed in a 250-mL conical flask with 30 mL Milli-Q water, and sonicated for 30 min (3 cycles of 10 min each) to yield a PM extract. The extract was filtered through six layers of sterile gauze, lyophilized to powder, and stored at −80 °C. For all extraction procedures, field blanks were included to ensure no extractable compounds from filters were present in the suspension to produce an effect beyond that noted with Milli-Q water suspension alone.

**Fig. 1.**
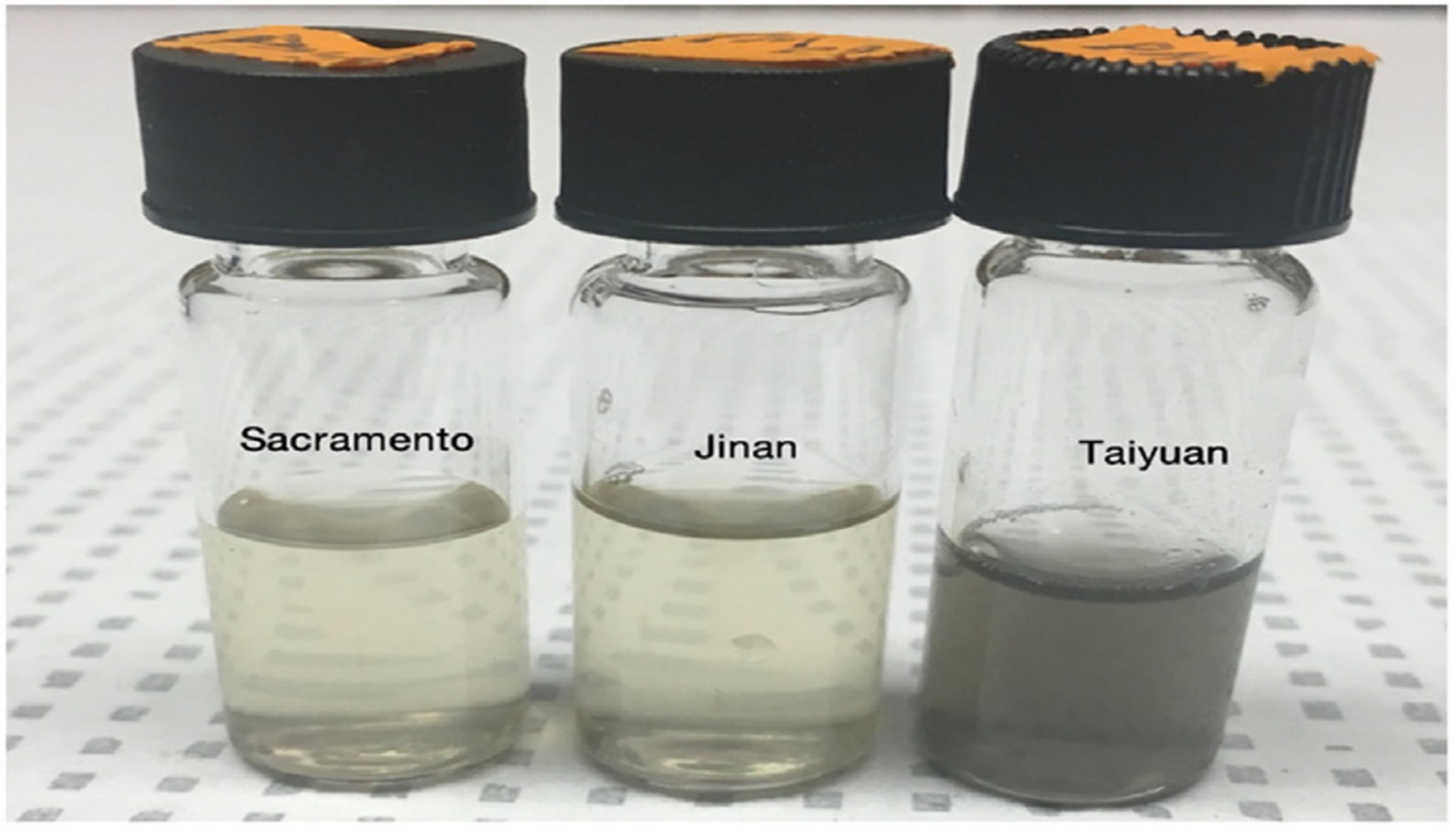
PM samples at equal mass concentrations. The image shows stock extracts of PM from Sacramento, California (left); Jinan, Shandong (middle); and Taiyuan, Shanxi (right) at a concentration of 1 μg/μL (1 mg/mL) suspended in nanopure water Milli-Q water. The PM sample from Taiyuan is strikingly darker in appearance.

Lyophilized PM extracts from Sacramento, Jinan, and Taiyuan were weighed, resuspended in Milli-Q water, and sonicated for 20 min to derive stock PM extract samples (PM_CA_, PM_SD_, and PM_SX_, respectively) of the same concentration (1 μg/mL; Fig. 1). All stock PM samples were frozen at −20 °C until needed.

### 2.3. Characterization of the hydrodynamic particle size

Dynamic light scattering (DLS) was used to determine the hydrodynamic particle size distribution of each stock extract sample following 20 min of sonication to insure adequate dispersion of particles (Sun et al., 2017).

### 2.4. Chemical characterization of stock extract samples via high-resolution time-of-flight aerosol mass spectrometry (HR-AMS)

Chemical characterization of all three PM extracts was performed at the University of California, Davis. Frozen stock PM extracts were thawed to 4 °C, sonicated for 20 min for thorough PM dispersal, and diluted by Milli-Q water to 0.1 μg/mL. Subsequently, 1 mL of each diluted stock sample was individually passed through a constant output aerosol generator with an inert argon carrier gas, and a silica gel diffuser where the sample became atomized. HR-AMS was used to determine the chemical composition of each extract sample. Chemical species such as nitrate, sulfate, chloride, ammonium, and organic compounds were quantified (Canagaratna et al., 2007). The HR-AMS spectra of organic compounds were further analyzed to determine PAH contents and associated elemental (hydrogen, carbon, oxygen, and nitrogen) composition and average degree of oxidation as detailed by Aiken et al. (2008), Ghio and Devlin (2001), Sun et al. (2017), Zhang et al. (2018), Yuan et al. (2020) and Dzepina et al. (2007).

### 2.5. Animals and exposure

The UC Davis Institutional Animal Care and Use Committee approved all procedures and animal housing practices. All methods in this study were carried out following relevant guidelines and regulations. As BALB/c mice exhibit strong T-helper (Th)-2-mediated responses that facilitate the detection of asthmatic and other inflammatory responses driven by PM (Li et al., 2009), 63 male, six-week-old BALB/c mice were purchased (Envigo, Hayward, CA) and randomly assigned to one of four groups for exposure to 1) vehicle control (Milli-Q water; n = 9), 2) PM_CA_ (n = 18), 3) PM_SD_ (n = 18); or 4) PM_SX_ (n = 18). All mice were acclimated for 2 weeks before the start of the exposure study, maintained on a 12-h light/dark cycle, and housed three per cage using sterile laboratory bedding and access to food and water ad libitum.

**Fig. 2.**
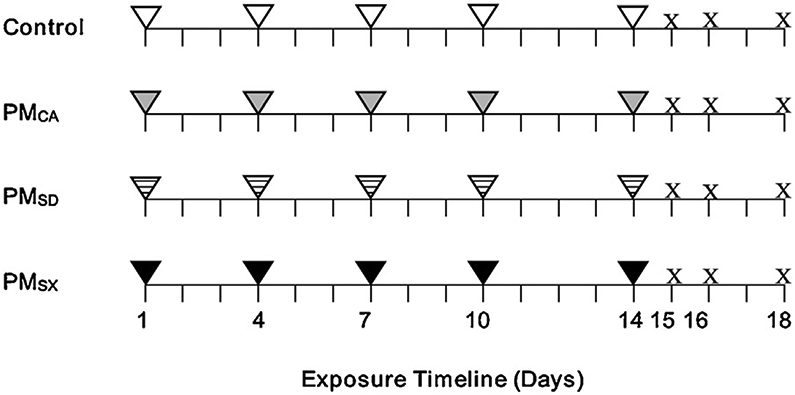
Exposure design timeline. The experimental schedules for four different mouse treatment groups are shown, with the x-axis representing the exposure timeline (days). The aspirates were 50 μL Milli-Q water (control; white triangles), or a 20μg/50 μL PM extract from Sacramento, California (gray triangles); Jinan, Shandong (striped triangles); or Taiyuan, Shanxi (black triangles). Each triangle in the figure represents a single exposure on days 1, 4, 7, 10, or 14. Each “X” represents day 1, 2, or 4 post OPA (shown as days 15, 16, or 18, respectively), when the euthanasias and collection of biological samples occurred. A total of 3 controls and 6 PM-exposed mice were euthanized on each necropsy day.

Immediately before exposure, stock extracts of PM_CA_, PM_SD_, and PM_SX_ were thawed, sonicated for 20 min, and diluted with Milli-Q water to a concentration of 20 μg/50 μL. Exposure by OPA occurred five times, once per day on days 1, 4, 7, 10, and 14 (Fig. 2). During exposure, mice were sedated by inhaling isoflurane in oxygen at a 3:1 ratio (Plummer et al., 2015). Each mouse was given 50 μL of Milli-Q water, or 20 μg of PM_CA_, PM_SD_, or PM_SX_ in 50 μL Milli-Q water via OPA such that the exposure volume was consistent across all groups. A single dose of this nature has been used previously (Sun et al., 2017) to examine PM effects. With high ambient PM levels noted in each of the sampled cities (in particular China), a total PM dose of 100 μg administered over two weeks could approach levels experienced by inhalation of ambient air over the same period.

### 2.6. Bronchoalveolar lavage fluid (BALF)

Mice were euthanized for necropsy at 1, 2, and 4 day(s) after the final OPA exposure (study days 15, 16, and 18, respectively; Fig. 2) with a 0.2-mL intraperitoneal injection of Beuthanasia-D pentobarbital solution (65 mg/kg body weight; Nembutal, Cardinal Health, Sacramento, CA). In the present study, the term “necropsy” refers to the post-mortem examination and collection of biological tissues from study animals. Each mouse was cannulated intratracheally, and the left mainstem bronchus was clamped, while the right lung was lavaged with two aliquots (0.6 mL/aliquot) of Dulbecco’s Phosphate Buffered Saline (PBS; Sigma-Aldrich, St Louis, MO). The collected BALF was centrifuged at 500 × g at 4 °C for 15 min to pellet the cells. BALF supernatant was decanted, frozen in liquid nitrogen, and stored at −80 °C. Cell pellets were resuspended in 500 mL PBS to determine total cell numbers and viability using a hemocytometer and 0.4 % Trypan Blue solution (Sigma-Aldrich).

**Fig. 3.**
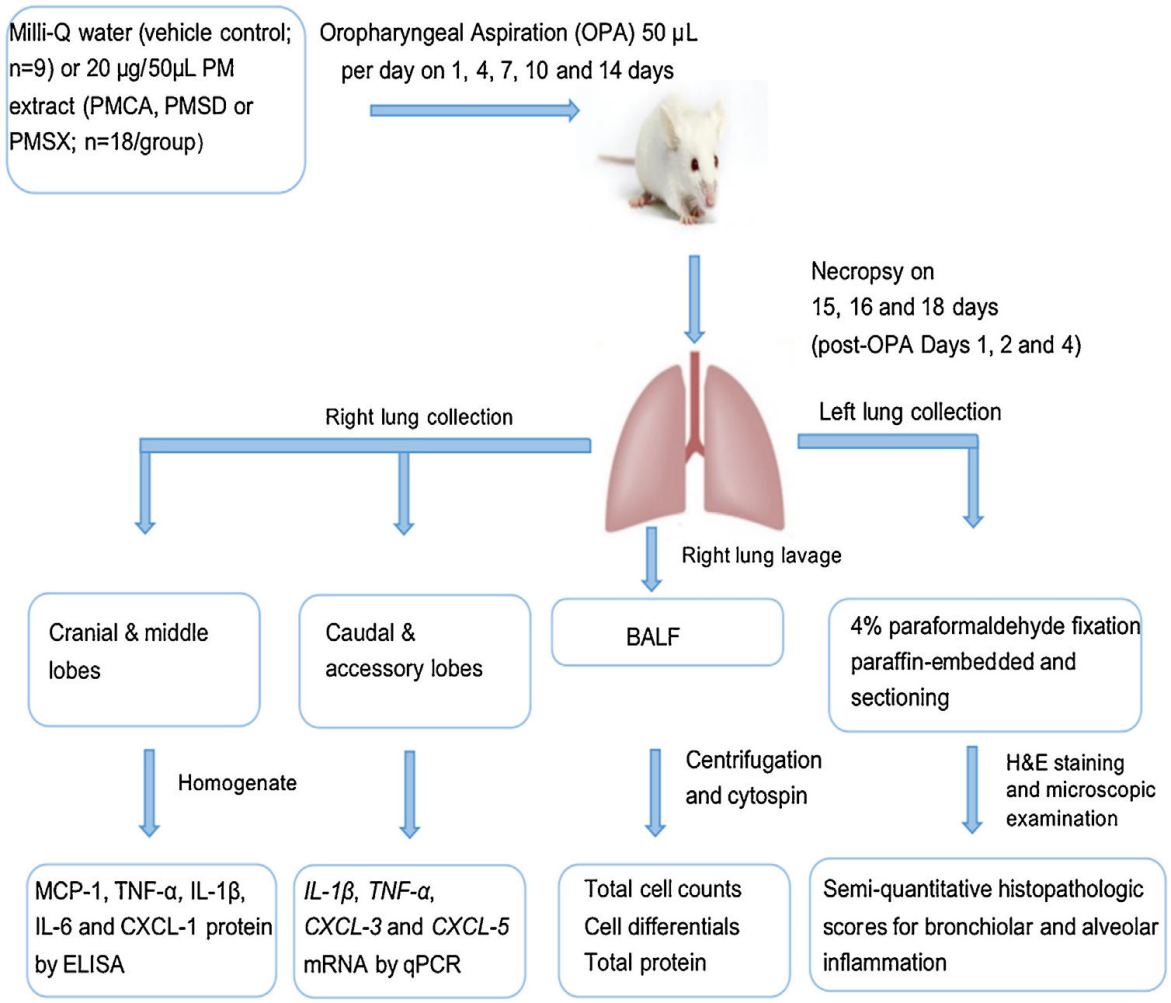
Experimental protocol. Four different treatment groups are shown along with their respective oropharyngeal aspiration exposures and post-necropsy processing.

BALF cytospin slides were prepared using a Shandon Cytospin (Thermo Shandon, Inc., Pittsburg, PA). The slides were then stained with DippKwik Differential Stain (American MasterTech, Lodi, CA) to determine the proportions of BAL macrophages, neutrophils, eosinophils, and lymphocytes in cell counts (500 cells/animal) performed using brightfield microscopy. The experimental protocol is shown in Fig. 3.

### 2.7. Lung tissue collection

The left lungs were inflated-fixed at 30 cm of pressure with 4% paraformaldehyde for one hour, stored in 4% paraformaldehyde for 48 h, and subsequently transferred into 70 % ethanol for later tissue processing for histopathology. The right lung lobes of each mouse were placed in two cryovials—one vial for the cranial and middle lobes—and the other for the caudal and accessory lobes. All vials were stored at −80 °C until further use.

### 2.8. Semi-quantitative lung histopathology

Following placement of the left lung in 70 % ethanol, four transverse slices (levels) were prepared as a method to sample uniformly histological changes throughout the entire lobe. These tissue slices were dehydrated, embedded in paraffin, and sectioned at a thickness of 5 μm. The sections were placed on glass slides, stained with Harris hematoxylin and eosin (H&E; American MasterTech, Lodi, CA), and coverslipped.

Four transverse slices were stained for each mouse (n = 9/control or 18/PM group). All slides were examined independently by two blinded observers (WY and SCV) for the presence of inflammation, cellular infiltrates, and cellular/tissue remodeling (i.e., desquamation and squamous metaplasia of airway epithelial cells, septal wall thickening) in alveolar ducts and airways using a semi-quantitative scoring scale. Previously published rubrics (Silva et al., 2013) were used to rank the severity (absent to marked; 0–3) and extent (0: no changes, 1: less than one-third of the slide, 2: one-half of the slide, 3: two-thirds of the slide) of the observed alveolar and bronchiolar inflammation. Alveolar inflammation involves the lung parenchyma, which includes the alveolar airspaces and pulmonary interstitium (i.e., the collection of support tissues within the lung that includes the alveolar epithelium, pulmonary capillary endothelium, basement membrane, and perivascular and perilymphatic tissues). With alveolar inflammation, focal accumulations of inflammatory cells composed of macrophages and/or polymorphonuclear cells (PMNs), e.g., neutrophils and eosinophils, can fill the alveolar airspace. Bronchiolar inflammation involves the airways, which can include the influx of PMNs and/or phagocytes (e.g., macrophages) in the airway wall and contiguous alveoli, as well as signs of airway cell damage (e.g., sloughing). For each mouse, the final score for a given parameter (e.g., alveolar inflammation) was the averaged product of the severity and extent scores for the four transverse slices. The use of the products resulted in scores ranging from 0 to 9, thus increasing the probability of finding significant (*p* < 0.05) differences between groups (Rutgers et al., 2010). Detailed semi-quantitative scoring guidelines are shown in Supplementary Table S1.

### 2.9. Gene expression analysis

As ribonucleic acids (RNAs) are the initial products of gene expression, messenger RNA (mRNA) was isolated from the right caudal and accessory lung lobes using TRI Reagent (Sigma-Aldrich) and a Quick-RNA Miniprep kit (Zymo Research, Irvine, CA). The mRNA was converted to complementary deoxyribonucleic acid (cDNA) using a high-capacity cDNA Reverse Transcription Kit (Applied Biosystems, Indianapolis, IN). Gene-specific mouse primers (0.2 μM; IDT, Coralville, IA), cDNA (2 μL/reaction), and SYBR Green (Applied Biosystems) DNA-binding stain were used for quantitative polymerase chain reaction (qPCR) measurements. Expression of genes for inflammatory cytokines, interleukin-1 beta (*IL-1β*) and tumor necrosis factor-alpha (*TNF-α*), and neutrophil chemokines, chemokine (C-X-C Motif) ligands-3 and −5 (*CXCL-3* and *CXCL-5*) were examined. Expression was assessed using the ΔΔ-Ct method and standardized to the expression of elongation factor 1-alpha 1 (*EEF1a1*) “housekeeping genes” (Castaneda et al., 2018). Housekeeping genes are 1) typically constitutive (i.e., continuously expressed); 2) required for maintenance of basic cellular functions essential for the existence of a cell irrespective of its role in tissue or the organism; and 3) expressed in all cells of an organism under normal and pathophysiological conditions (Pampel, 2017). The *EEF1a1* gene is one of the alpha subunit forms of the elongation factor 1 complex that interacts with aminoacylated transfer RNA (tRNA) and delivers it to the A site of the ribosome during the elongation phase of protein synthesis. It is constitutively expressed in all tissues in mice and humans, eukaryotes in general (Gentile et al., 2016). The use of *EEF1a1* genes is an accepted method for qPCR gene expression analysis of mouse lung tissue (Castaneda et al., 2018).

Mouse gene primers were designed using Primer3 primer design software (Untergasser et al., 2012). Primers used in this study are detailed in Supplementary Table S2.

### 2.10. Enzyme-Linked immunosorbent assay (ELISA)

ELISAs (Biolegend, San Diego, CA) were performed on the homogenized cranial and middle lobes of the right lung to analyze concentrations of specific proteins, including TNF-α; monocyte chemoattractant protein-1 (MCP-1), a chemoattractant responsible for monocyte and macrophage recruitment; CXCL-1, seen in inflammation or wound healing; and IL-1β and IL-6, both mediators of inflammatory responses. The cranial and middle lobes from control- and PM-exposed animals and standards from the R&D Systems ELISA kits (1000 μg/mL to 7.8 μg/mL) were prepared and examined in duplicate in 96-well plates using a SpectroMax plate reader (Molecular Devices, Sunnyvale, CA). Duplicate readings were averaged. All concentrations were normalized to total lung protein and reported in pg of specific protein per mg of lung tissue.

### 2.11. Statistical analysis

No data points were excluded before statistical analysis. All statistical tests were performed using GraphPad PRISM 8.0 software. A value of *p* < 0.05 was considered statistically significant. For each measured endpoint, a Shapiro-Wilk test was first used to detect normality, and a one-way analysis of variance (ANOVA) and *post hoc* Tukey’s test were performed to determine differences due to treatment. All data in the present document are expressed as the mean ± standard error of the mean (SEM).

## 3. Results

### 3.1. DLS particle size analysis

**Fig. 4.**
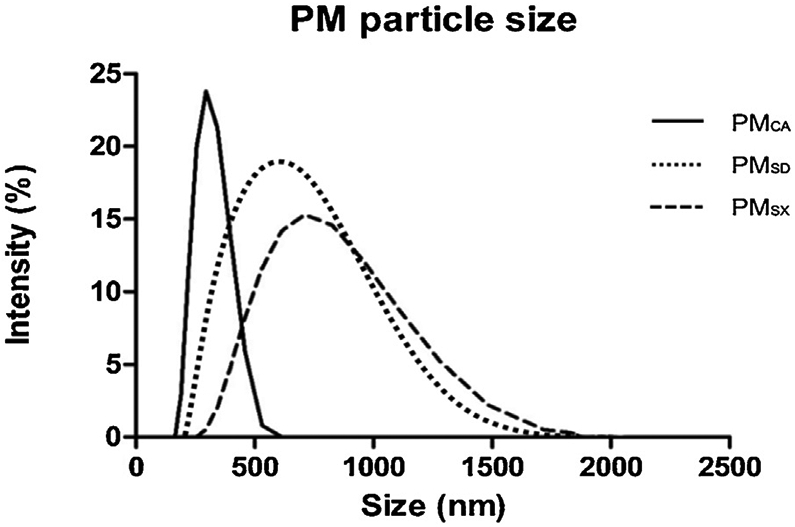
Hydrodynamic particle size distributions of wintertime PM extracts from Sacramento, California (PM_CA_); Jinan, Shandong (PM_SD_); and Taiyuan, Shanxi (PM_SX_). The graph shows the results from dynamic light scattering analyses. Particle density (intensity %) is shown on the y-axis as a function of particle diameter (size on the x-axis) in nanometers.

All stock PM extracts exhibited unimodal hydrodynamic size distributions. PM_CA_ ranged between 100–600 nm, while PM_SD_ and PM_SX_ ranged between 100 nm–1800 nm (Fig. 4). Based on the hydrodynamic diameters measured by DLS, all PM samples were within the PM_2.5_ size range in their hydrodynamic measured diameters (Fig. 4).

### 3.2. Chemical composition of PM extracts

**Fig. 5.**
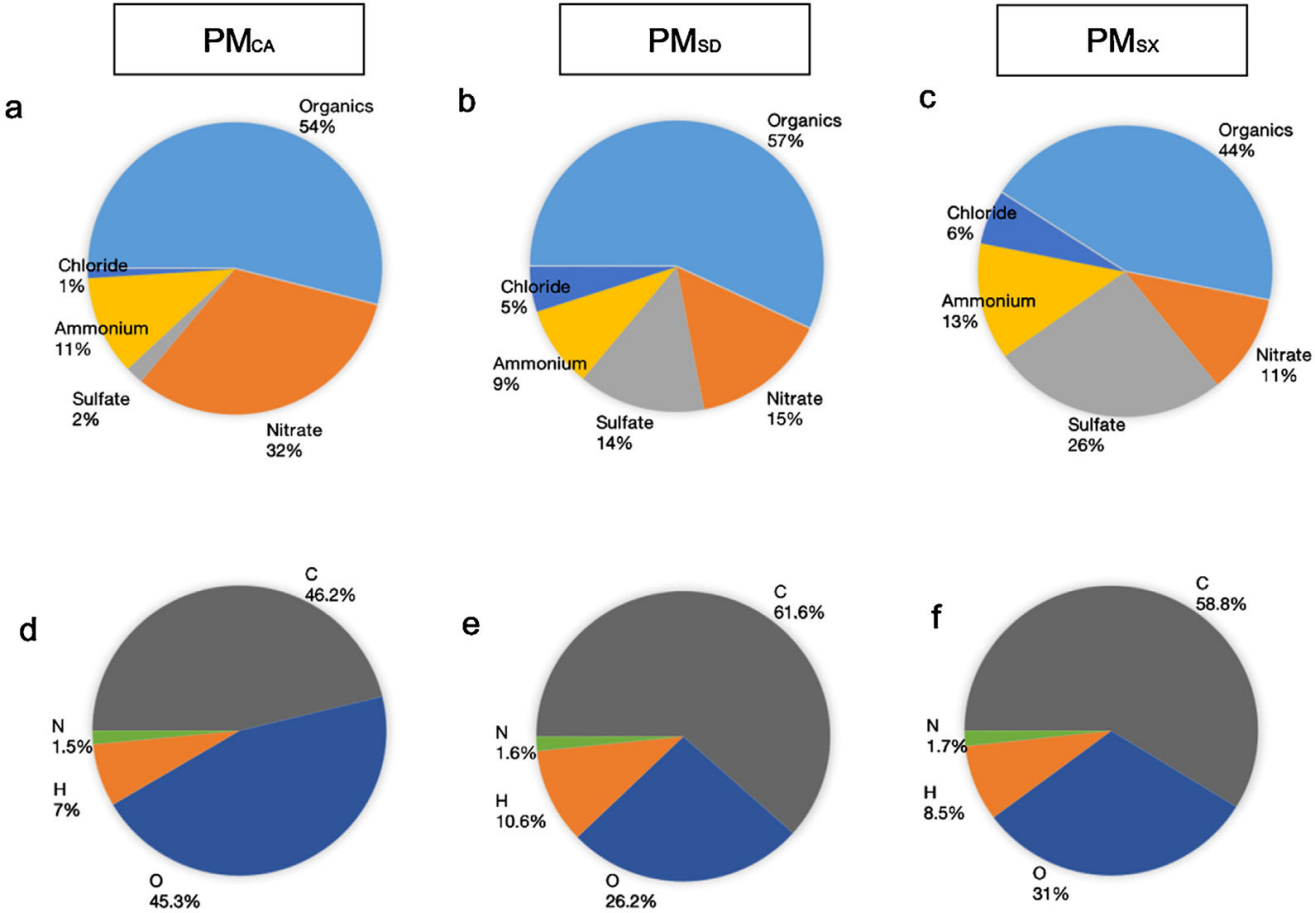
Chemical composition of wintertime PM extracts from Sacramento, California; Jinan, Shandong; and Taiyuan, Shanxi. Characterization was performed using high-resolution time-of-flight aerosol mass spectrometry. Charts (a-c) in the top row illustrate bulk composition, including organic matter. Charts (d-f) in the bottom row show the average elemental composition of the organic matter. Abbreviations: C- carbon; H – hydrogen; N – nitrogen; O – oxygen.

Organic compounds comprised the largest fraction of the total solute mass in each extract sample. While the PM_SX_ extract was the darkest (Fig. 1), suggesting it had the highest black carbon content, PM_SD_ was also found to contain carbon. Organic compounds accounted for 54 %, 57 %, and 45 % of the PM_CA_, PM_SD_, and PM_SX_ mass, respectively (Fig. 5a-c). These results based on the color and chemical analysis of each PM extract suspension suggests the forms of carbon are different, at least with respect to the PM_SX_ and PM_SD_ extracts. Within the organic fraction, carbon and oxygen accounted for at least 87 % of the mass (Fig. 5d-f). For other compounds measured by HR-AMS, nitrate and sulfate fractions were highly variable, while those of chloride and ammonium were relatively similar among the California and China PM extracts. Nitrate was more abundant in California PM than China PM, while sulfate was most prevalent in PM_SX_ versus the other two PM extracts (Fig. 5a-c). The sulfate and nitrate measured in all three PM samples were principally inorganic, identified by the presence of anions in both the ammonium sulfate and ammonium nitrate. However, based on ion balance analysis and the abundance of sulfur-containing organic ions in the HR-AMS spectrum, it is likely that PM_SX_ contained more organic sulfate than the other two PM extracts, perhaps a reflection of sulfur-containing coal combusted locally. Of the PAH levels analyzed by HR-AMS, the PAH/Organics ratios were 0.0017, 0.004, and 0.0024 of the PM_CA_, PM_SD_, and PM_SX_, respectively. The percentage of dissolved PM mass varied between samples due to the different organic fractions. Therefore, based on the calculated enhancement factors relative to PM_CA_, PM_SD_ and PM_SX_ had 2.48 and 11.50 times higher levels than PM_CA_, respectively (Table 1).

### 3.3. Cell differentials in BALF

**Fig. 6.**
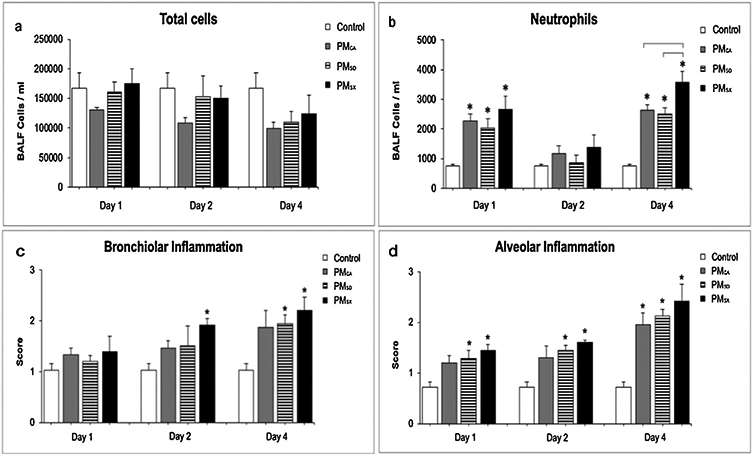
Oropharyngeal aspiration of particulate matter (PM) extracts produced elevated BALF neutrophil numbers and tissue inflammation in mice up to four days post exposure. Animals were exposed on five separate days over two weeks to Milli-Q water (Control), or a PM extract (20 μg/50 μL) from Sacramento, CA; Jinan, Shandong; or Taiyuan, Shanxi (PM_CA_, PM_SD_ or PM_SX_ respectively). N = 9 for controls, and 18/PM group (6/group/day). Each mouse received a 50-μL aspirate on the exposure days (total PM dose = 0 or 100 μg/mouse), and necropsies occurred on day 1, 2, or 4 post exposure. Panels a-b show counts of total bronchoalveolar lavage fluid (BALF) cells and neutrophils, respectively. Panels c-d show semi-quantitative histopathology scores from the bronchiolar and alveolar lung regions, respectively. Final histopathology scores were averaged from the products of the severity and extent scores in each region of the lungs. Data from different time points in each panel were analyzed separately via one-way ANOVAs and presented as the mean ± standard error of the mean. Asterisks (*) indicate significant (*p* < 0.05) differences from control. Brackets indicate significant (*p* < 0.05) differences between PM-exposed groups.

The BALF cell analyses were based on a total of 9 control mice (n = 3/time-point) and 54 PM-exposed mice (n = 6/group/time-point). A total of 9 control mice pooled from post-OPA days 1, 2, and 4 were used for comparisons since there were no statistical differences among controls over time. Recovery time did not appear to play a role in the responses observed in BALF as there were no statistically significant differences between necropsy days (Fig. 6a-b). No significant (*p* < 0.05) exposure-related differences were noted for total cell counts of mice exposed to the vehicle control versus PM_CA_, PM_SD_, or PM_SX_ irrespective of the recovery time post-OPA (Fig. 6a). In contrast, significantly increased neutrophil numbers were observed on post-OPA days 1 and 4 in all PM-exposed groups relative to controls (*p* < 0.05 for all comparisons), and on day 4 in PM_SX_-exposed mice relative to their PM_CA_- and PM_SD_-exposed counterparts (*p* = 0.0326 and *p*= 0.0196, respectively; Fig. 6b). No statistically significant treatment-related effects were observed on day 2 (Fig. 6b), as neutrophil numbers in all PM-exposed groups dropped temporarily to near-control levels. However, the elevated neutrophil numbers observed in PM- versus control-exposed mice on day 4 is not unlike results of previous studies (Noel et al., 2016; Smith et al., 2006) in which incomplete lung recovery—including elevated BALF neutrophils and other measures of inflammation— has been reported in mice and rats exposed sub-acutely to combustion-derived PM and examined up to 10 days post exposure.

### 3.4. Histological analysis

**Fig. 7.**
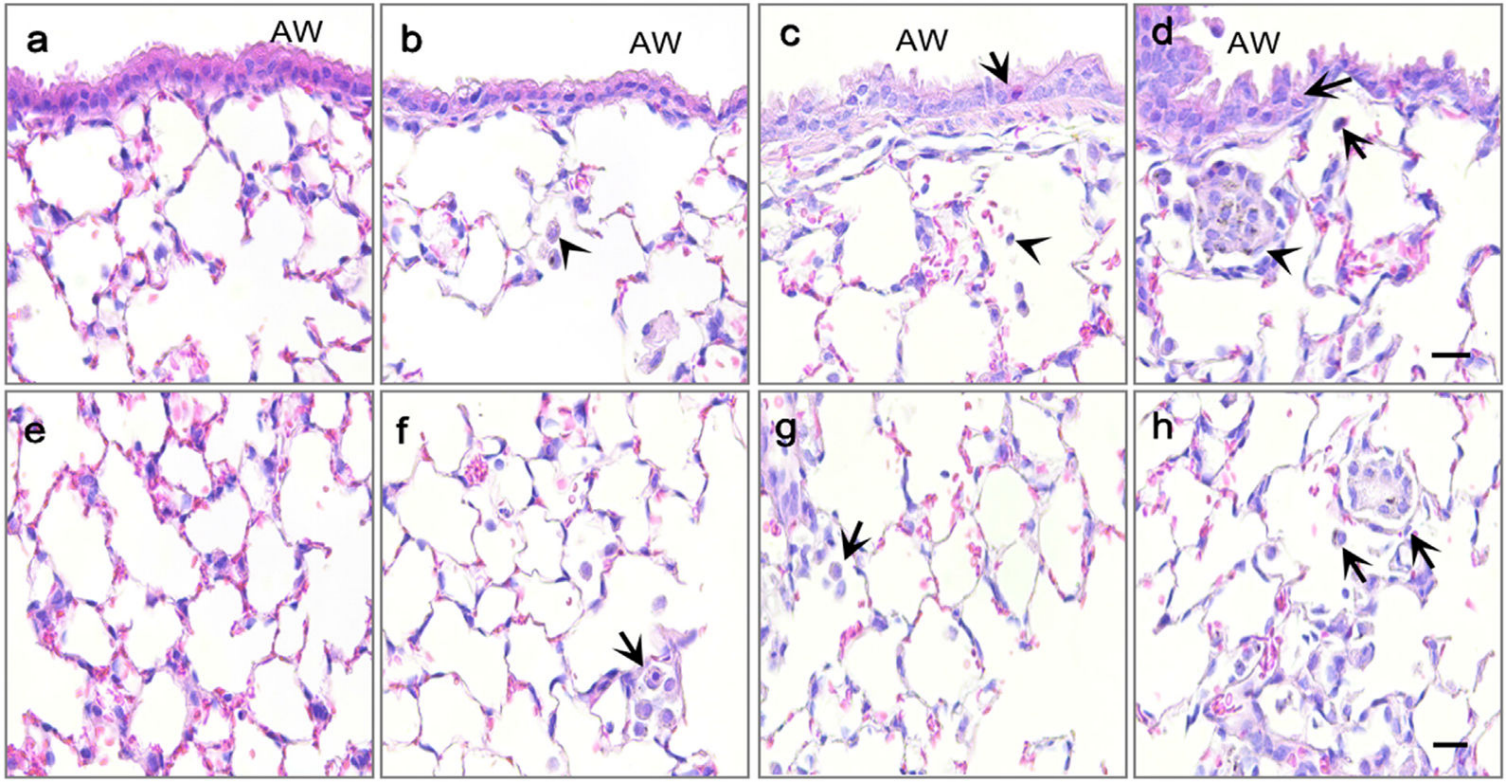
Lung tissue inflammation in mice exposed to particulate matter (PM) extracts. Panels are light micrographs of hematoxylin- and eosin-stained bronchiolar (a-d) and alveolar (e-h) tissues collected on day 4 after the last of five separate 50-μL oropharyngeal aspiration exposures to a MilliQ water control (a & e; n = 9) or a 20-μg/50 μL PM extract (n = 18/group) from Sacramento, CA (b & f); Jinan, Shandong (c & g); or Taiyuan, Shanxi (d & h). The total PM dose was 0 or 100 μg/mouse over 14 days. In panels a-d, arrows indicate neutrophils, and arrowheads indicate macrophages. In panels e-h, arrows identify regions of alveolar inflammation. AW = airway, scale bar is 20 μm.

There were no significant (*p* < 0.05) differences in bronchiolar inflammation scores on post-OPA day 1 (Fig. 6c). However, on day 2, mice exposed to PM_SX_ exhibited significantly (*p* = 0.0179) more bronchiolar inflammation than controls (Fig. 6c). By day 4, both groups given Chinese PM extracts exhibited more severe bronchiolar inflammation than controls (*p* < 0.04 for PM_SD_; *p* < 0.01 for PM_SX_; Fig. 6c), with neutrophilic influxes into the peribronchiolar and alveolar regions of the lungs that were not observed in other groups (Fig. 7a-d). Mice exposed to PM_SX_ were found to have visible black particles—likely due to coal combustion—in macrophages throughout the alveolar regions of the lungs.

In contrast to the bronchiolar, perivascular, and subpleural regions, the alveoli appeared to be most affected by PM. On post-OPA days 1 and 2, significantly greater alveolar inflammation was observed in groups administered PM_SD_ (*p* = 0.0206 and 0.0361, respectively) or PM_SX_ (*p* = 0.0057 and 0.0172, respectively) versus the vehicle control (Fig. 6d). Over these two days, no significant intra-group differences in alveolar inflammation were observed within the PM_SD_ or PM_SX_ groups. By day 4, all three PM-exposed groups exhibited higher scores for alveolar inflammation than controls (Fig. 6d; *p* < 0.01, *p* < 0.01, and *p* < 0.0001 for PM_CA_, PM_SD_, and PM_SX_, respectively). Mice exposed to PM_SD_ and PM_SX_ had average scores of 1.8 and 2.4, respectively, for alveolar inflammation, with notable changes in alveolar wall thickening, and numerous free macrophages distributed throughout the parenchyma (Fig. 7e-f), while control mice had an average score of 0.7, with thin-walled alveolar septa and relatively few lumenal macrophages (Figs. 6d and 7e). Although inter-group differences were not statistically significant among the PM-exposed mice, those administered PM_SX_ appeared to demonstrate the greatest effects (Fig. 7e-f) with cellular debris and numerous foamy, particle-laden macrophages aggregated in the alveolar airspaces (Fig. 7f). Trends toward greater but not statistically significant bronchiolar and alveolar inflammation in the PM-exposed groups over time (Fig. 6c-d) support the aforementioned findings of elevated BALF neutrophils in these groups relative to controls on post-OPA day 4 (Fig. 6b), and the likelihood of unresolved and/or late-peaking inflammation with repeated exposure to combustion-derived PM.

### 3.5. qPCR analysis of gene expression

**Fig. 8.**
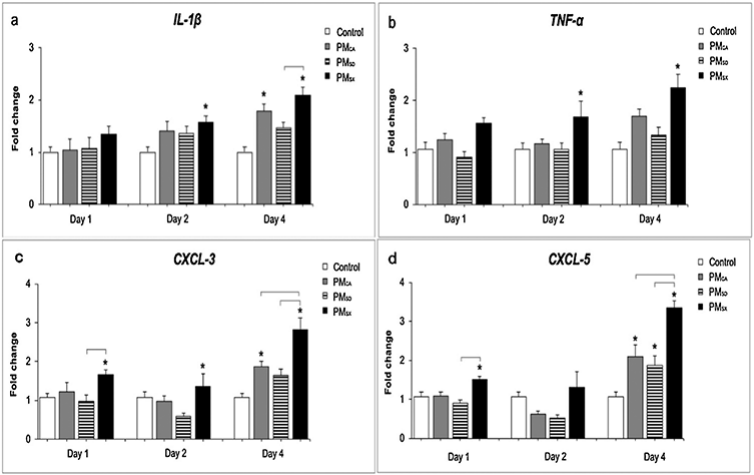
Extracts of particulate matter (PM) from Taiyuan, China increased expression of genes for the inflammatory cytokines, Interleukin (*IL*)-*1β* and Tumor Necrosis Factor (*TNF-α*; panels a-b) and for C-X-C motif ligands-3 and −5 (*CXCL-3* and −5; panels c-d) which affect neutrophil mediation of monocyte chemotaxis. Graphs show results from quantitative polymerase chain reaction assays performed on mRNA obtained from the lungs of mice at 1, 2, or 4 day(s) after the last 50-μL oropharyngeal aspiration (OPA) exposure to MilliQ water, or a PM extract (20 μg/50 μL) from Sacramento, CA (PM_CA_); Jinan, Shandong (PM_SD_); or Taiyuan, Shanxi (PM_SX_). N = 9 for controls, and 18/PM group (6/group/day). OPA occurred on five separate days over two weeks, yielding a total PM dose of 0 or 100 μg/mouse. Gene levels were analyzed for each animal and averaged for each treatment group. Gene expression is shown relative to the housekeeping gene, *EEf1a1*. Data are shown as the mean ± SEM. One-way ANOVA and Tukey tests were performed at a significance level of *p* < 0.05. Asterisks (*) indicate significant (*p* < 0.05) differences from vehicle control; brackets indicate significant (*p* < 0.05) differences between non-control groups.

PM-induced recruitment of neutrophils into the lungs can be directed by numerous chemotactic mediators, including *IL-1β*, *TNF-α*, *CXCL-3*, and *CXCL-5* (Maueroder et al., 2015). Results demonstrated expression of *IL-1β* and *TNF-α* genes was significantly increased in PM_SX_- versus control-exposed mice on days 2 (*p* = 0.0261 and *p* = 0.0415, respectively) and 4 (*p* < 0.0001 and *p* = 0.0062, respectively; Fig. 8a-b) post OPA. Similar increases were observed for post-OPA day 4 *IL-1β* in PM_CA_-exposed mice relative to controls (*p* = 0.0049) and in PM_SX_-exposed mice relative to their PM_SD_-exposed counterparts (*p* = 0.0152; Fig. 8a-b). Gene expression of *CXCL-3* and *CXCL-5* was also significantly elevated in PM-versus control-exposed mice, with PM_SX_ producing increases in one or both chemokines at all post-OPA time points (*p* < 0.05); PM_CA_ producing increases in *CXCL-3* (*p* = 0.0351) and *CXCL-5* (*p* < 0.0001) on post-OPA day 4 alone; and PM_SD_ producing an increase in *CXCL-5* (*p* = 0.0233) on post-OPA day 4 alone (Fig. 8c-d). *CXCL-3* and/or −5 mRNA expressions increased in all PM- versus control-exposed groups on post-OPA day 4.

### 3.6. Quantification of cytokines by ELISA

**Fig. 9.**
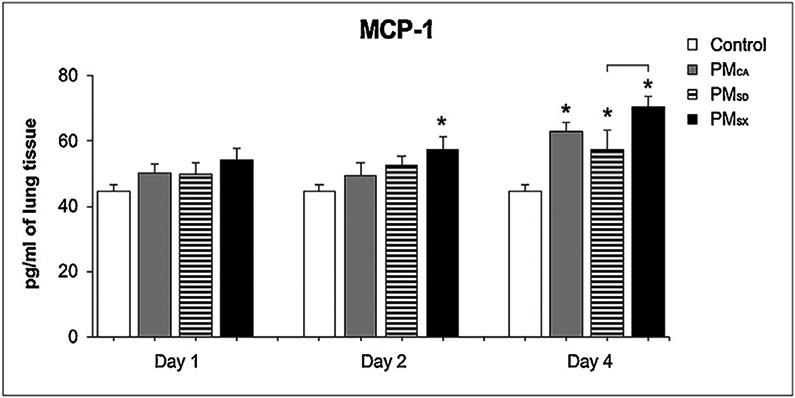
Mice exhibited subacute increases in Monocyte Chemoattractant Protein (MCP)-1 levels following exposure to particulate matter (PM) extracts from California or China. The bar graph shows the results from enzyme-linked immunosorbent assays performed on lung tissues from mice at 1, 2, or 4 day(s) after the last 50-μL oropharyngeal aspiration (OPA) exposure to MilliQ water (Control), or a PM extract (20 μg/50 μL) from Sacramento, CA (PM_CA_); Jinan, Shandong (PM_SD_); or Taiyuan, Shanxi (PM_SX_). N = 9 for total controls, and 6/post-OPA day (1, 2, and 4) for each PM. Over two weeks, OPA occurred on five separate days, yielding a total PM dose = 0 or 100 μg/mouse. Separate ANOVAs were performed for each time point to determine inter-group differences due to exposure. Asterisks (*) indicate significant (*p* < 0.05) differences from control; brackets indicate a significant (*p* < 0.05) difference between PM_SD_ and PM_SX_.

Although six cytokines were examined, concentrations of three proteins (TNF-α, CXCL-1, and IL-6) were too low to quantify reliably with limits of detection at 5.16, 66.521, 8.301 ng/mL, respectively. Of the two remaining cytokines, IL-1β (data not shown) and MCP-1 (Fig. 9), only protein levels of the latter varied significantly among the PM- and control-exposed groups, with PM_SX_ producing increases relative to control on post-OPA days 2 and 4 (*p* < 0.05 and *p* < 0.0001, respectively); and PM_CA_ and PM_SD_ producing similar increases on post-OPA day 4 alone (*p* < 0.05 and *p* < 0.05, respectively).

## 4. Discussion

Global PM emissions are of major concern, with the duration of PM exposure and physicochemical characteristics of PM (e.g., diameter and composition) among the primary factors influencing adverse health effects. Of the various PM size fractions, PM_2.5_ is most associated with morbidity and premature mortality (Rajak and Chattopadhyay, 2019) after long-term exposure, and, because it contributes to 7 million deaths each year (WHO, 2019), PM_2.5_ is often described as one of the leading risk factors for premature mortality. Though mortality was not examined in the present study, cumulative results add to the existing body of literature regarding 1) the impacts of repeated seasonal PM_2.5_ exposure on pulmonary morbidity, and 2) the variability of PM_2.5_ toxicity related to chemical composition.

The prior two studies performed in our laboratory (Sun et al., 2017; Yuan et al., 2020), with similar PM extracts administered in single-exposure paradigms, showed striking differences in findings compared to the current study with repeated PM exposure of different duration and frequency. In the present study, many of the statistically significant responses were observed on post-OPA day 4, with BAL (Fig. 6b), histopathology (Fig. 6c-d), gene expression (Fig. 8), and ELISA (Fig. 9) analyses. Less severe inflammatory responses were noted on days 1 and 2. The cumulative findings suggest repeated exposure to PM_CA_, PM_SD_, or PM_SX_ was associated with significant and persistent inflammation relative to the sham controls. These results are in striking contrast to those from our previous study (Yuan et al., 2020), which measured the inflammatory effects of a single 50-μL OPA exposure to Milli-Q water or a PM_CA_, PM_SD_, or PM_SX_ stock (1 μg/μL) extract on post-OPA days 1, 2 and 4. In that study, the three PM extracts produced the most severe inflammatory responses on post-OPA day 1, with all measured biological parameters returning to control levels by days 2 and 4. Given the identical animal model and PM extract composition for both studies, the drivers for different findings in the two studies are likely to be the 1) longer exposure duration, which stimulated inflammation pathways that are initiated subacutely; 2) intermittent, repeated exposure frequency that prevented resolution of the inflammatory response; 3) lower dose rate which could have prevented the strong acute inflammation response; and 4) higher total dose of PM delivered (100 μg versus 50 μg) in the present versus the previous (Yuan et al., 2020) study.

Several reviews have concluded the chemical components of PM can play an important role in post-exposure health effects (Chen and Lippmann, 2009; Rohr and Wyzga, 2012). Therefore, to enact the proper exposure controls (e.g., protective equipment, concentration limits), it is critical to determine which PM components, or combinations thereof, are most harmful to human health (Wyzga and Rohr, 2015). In the present study, repeated exposure to PM_SX_ frequently produced the greatest differences relative to control (Figs. 6,8, and 9). The combined findings for BALF neutrophil numbers and lung histopathology provided some confirmation that the chemical composition of PM_SX_ may contribute to greater toxicity than that observed for PM_CA_ and PM_SD_ administered on an equal PM mass basis. Differences between the PM-exposed groups were most likely due to differing chemical compositions influenced by regional emission sources such as vehicular exhaust, agricultural and industrial operations in the Sacramento region, or coal production in the Shandong and Shanxi provinces (Plummer et al., 2015; Xia et al., 2013).

Although we were unable to definitively identify the precise chemical constituent(s) that produced the observed biological effects, it appears that organosulfates and polyaromatic hydrocarbons (PAHs) likely contributed to the differential induction of biological effects observed in the present study (Table 1). Sulfate levels measured at 2%, 14 %, and 26 % in PM_CA_, PM_SD_, and PM_SX_, respectively (Fig. 5a-c). Several studies (Chen and Lippmann, 2009; Krewski et al., 2005) have indicated sulfate-associated particles (i.e., fossil fuel combustion products) are among the most toxic in terms of cardiopulmonary and cardiovascular disease effects and lung cancer mortality. Particulate air pollution, characterized by high secondary aerosol concentrations, including organosulfates, has been a serious environmental problem during recent winters in China (Huang et al., 2018; Wang et al., 2017). Thus, higher fractions of organosulfates are plausible for PM_SX_ and PM_SD_ compared to PM_CA_. However, in the present study, speciation of sulfates was not possible; therefore, future exposure studies could benefit from quantifying organic and inorganic sulfates as a means to understand better the health risks they pose.

All three PM extracts used in the current study were found to activate the aryl hydrocarbon receptor (AhR), as well as *CYP1A1* mRNA expression in human HepG2 cells (Yuan et al., 2020). These effects are most likely due to PAHs in the carbonaceous fraction of each PM extract. In the current research, compared to PM_CA_, the PAH levels were 2.48 and 11.5 higher in PM_SD_ and PM_SX_, respectively (Table 1). PAHs have received considerable attention due to their potential toxic, carcinogenic, and mutagenic effects. Coal combustion is known to produce PAHs (Shen et al., 2010), and in 2012, China’s coal consumption was 2.75 billion tons, approximately one-half of the global coal consumption. Shanxi province is one of China's largest coal-producing centers (Yang and Teng, 2018).

Repeated exposure to PM_SX_ produced a higher BALF neutrophil count than PM_CA_ and PM_SD_ on post-OPA day 4 (Fig. 6b). Repeated exposure to PM has been shown to increase BAL neutrophils that may be causally associated with lung injury (Maueroder et al., 2015; Pardo et al., 2016), with the higher numbers of neutrophils entering the lungs typically reflective of the severity of the inflammatory and cellular response. Neutrophil influx was clearly observed in our study, with neutrophils present in the lung interstitium and among epithelial cells (Fig. 7a-d). Treatment groups in which neutrophil numbers in BALF attained a level of statistical significance also exhibited significantly greater histological inflammatory scores in the airways and alveoli on day 4 post OPA (Fig. 6c-d). Neutrophils are generally present in these two areas of the lungs during periods of inflammation.

In the present study, mice exposed to PM_SX_ exhibited significantly increased mRNA for *TNF-α* and *IL-1β*, cytokines involved in pulmonary inflammatory reactions (Li et al., 2017), on post-OPA days 2 and 4 (Fig. 8a-b), as well as *CXCL-3* and *CXCL-5* neutrophil chemokines (Bhatia et al., 2012; Narayanan et al., 2005) on post-OPA day 4 (Fig. 8c-d) when compared to control-, PM_CA_-, and PM_SD_-exposed groups. MCP-1 protein was increased in all PM-exposed groups relative to controls on day 4 post OPA, but PM_SX_ produced the longest-lasting response that was also stronger than that for PM_SD_ (Fig. 9). MCP-1 is a chemoattractant responsible for monocyte and macrophage recruitment. However, growing evidence shows MCP-1 may also be involved in attracting neutrophils (Balamayooran et al., 2011). When considered cumulatively, the concomitant elevation of cytokine (*TNF-α* and *IL-1β*) and chemokine (*CXCL-3*, *CXCL-5*) mRNA, MCP-1 protein, and BALF neutrophils on post-OPA day 4 suggest multiple chemical mediators may be driving high neutrophil numbers even after 4 days of recovery.

Findings from the present study provide further evidence for potential mechanisms by which PM extracts from different regions of the world promote time-lagged effects. Future studies could use later post-OPA time points to compare persistent pulmonary health effects of repeated exposure to extracts of PM_2.5_ and test the impact of elevated PM-sulfur on the degree of inflammation. These future studies would significantly add to the existing knowledge to correlate PM composition to pulmonary responses, inform future regulations on source-specific emissions, and better protect and benefit human health.

## 5. Conclusions

Consistent with universal time-lag effects of PM on health, the results in the current study showed exposure to PM_CA_, PM_SD_, or PM_SX_ produced greater toxicity on post-OPA day 4, compared to days 1 and 2. These results suggest sulfate and PAH content may have influenced the observed toxicity for PM_CA_, PM_SD_, and PM_SX_. Mice exposed to PM_SX_ demonstrated the greatest inflammatory responses, as evidenced by statistically significant (*p* < 0.05) increases in pulmonary neutrophil numbers and pro-inflammatory cytokines and chemokines that may be due to higher sulfate and PAH content, compared to that of PM_CA_ and PM_SD_. In summary, the current study provides compelling evidence that PM extracts from different regions promote adverse time-lagged health effects influenced by chemical composition. These findings further highlight the need to develop source-specific regulations to protect human health.

## Supplementary Material

supplement

## Figures and Tables

**Table 1:** HR-AMS-defined PAH levels in Sacramento, California (PM_CA_); Jinan, Shandong (PM_SD_); and Taiyuan, Shanxi (PM_SX_) particulate extract samples.

Sample	PAH/Organic ratio	Organic fraction	Percentage of dissolved PM mass (%)	Enhancement factor
PM_CA_	0.00017	0.54	0.00918	1.00
PM_SD_	0.0004	0.57	0.02280	2.48
PM_SX_	0.0024	0.44	0.10560	11.5

The method used to determine the total PAH-to-organic PM mass ratio (Table 1) by AMS was first described by Dzepina and colleagues (2007).

## HIGHLIGHTS

- Time-lagged effects were evaluated in mice following repeated exposures to one of three PM extract samples.
- All three PM extracts induced progressive inflammatory responses over time.
- These time-lagged effects were influenced by the chemical composition and potentially the PAH content of each PM extract.

## References

[R1] Aiken AC, Decarlo PF, Kroll JH, Worsnop DR, Huffman JA, Docherty KS, Ulbrich IM, Mohr C, Kimmel JR, Sueper D, Sun Y, Zhang Q, Trimborn A, Northway M, Ziemann PJ, Canagaratna MR, Onasch TB, Alfarra MR, Prevot AS, Dommen J, Duplissy J, Metzger A, Baltensperger U, Jimenez JL, 2008. O/C and OM/OC ratios of primary, secondary, and ambient organic aerosols with high-resolution time-of-flight aerosol mass spectrometry. Environ. Sci. Technol 42, 4478–4485. doi:10.1021/es703009q. 18605574

[R2] Anderson JO, Thundiyil JG, Stolbach A, 2012. Clearing the air: a review of the effects of particulate matter air pollution on human health. J. Med. Toxicol 8, 166–175. doi:10.1007/s13181-011-0203-1. 22194192 PMC3550231

[R3] Balamayooran G, Batra S, Balamayooran T, Cai S, Jeyaseelan S, 2011. Monocyte chemoattractant protein 1 regulates pulmonary host defense via neutrophil recruitment during Escherichia coli infection. Infect. Immun 79, 2567–2577. doi:10.1128/IAI.00067-11. 21518788 PMC3191985

[R4] Bein KJ, Wexler AS, 2015. Compositional variance in extracted particulate matter using different filter extraction techniques. Atmos. Environ 107, 24–34. doi: 10.1016/j.atmosenv.2015.02.026.

[R5] Bhatia M, Zemans RL, Jeyaseelan S, 2012. Role of chemokines in the pathogenesis of acute lung injury. Am.J. Respir. Cell Mol. Biol 46, 566–572. doi:10.1165/rcmb.2011-0392TR. 22323365 PMC3361356

[R6] Cadelis G, Tourres R, Molinie J, 2014. Short-term effects of the particulate pollutants contained in Saharan dust on the visits of children to the emergency department due to asthmatic conditions in Guadeloupe (French Archipelago of the Caribbean). PLoS One 9, e91136. doi:10.1371/journal.pone.0091136. 24603899 PMC3946322

[R7] Canagaratna MR, Jayne JT, Jimenez JL, Allan JD, Alfarra MR, Zhang Q, Onasch TB, Drewnick F, Coe H, Middlebrook A, Delia A, Williams LR, Trimborn AM, Northway MJ, DeCarlo PF, Kolb CE, Davidovits P, Worsnop DR, 2007. Chemical and microphysical characterization of ambient aerosols with the aerodyne aerosol mass spectrometer. Mass Spectrom. Rev 26, 185–222. doi:10.1002/mas.20115. 17230437

[R8] Castaneda AR, Vogel CFA, Bein KJ, Hughes HK, Smiley-Jewell S, Pinkerton KE, 2018. Ambient particulate matter enhances the pulmonary allergic immune response to house dust mite in a BALB/c mouse model by augmenting Th2- and Th17-immune responses. Physiol. Rep 6, e13827 doi:10.14814/phy2.13827. 30230272 PMC6144457

[R9] CDPH, 2016. Sacramento county asthma profile. In: health, C.d.o.p (Ed.), County Asthma Profiles, .

[R10] Chen LC, Lippmann M, 2009. Effects of metals within ambient air particulate matter (PM) on human health. Inhal. Toxicol 21, 1–31. doi:10.1080/08958370802105405. 18803063

[R11] Dzepina K, Arey J, Marr LC, Worsnop DR, Salcedo D, Zhang Q, Onasch TB, Molina LT, Molina MJ, Jimenez JL, 2007. Detection of particle-phase polycyclic aromatic hydrocarbons in Mexico city using an aerosol mass spectrometer. Int. J. Mass Spectrom 263, 152–170. doi:10.1016/j.ijms.2007.01.010.

[R12] EPA, 2017. How Does PM Affect Human Health? . https://www3.epa.gov/region1/airquality/pm-human-health.html.

[R13] Gentile AM, Lhamyani S, Coin-Araguez L, Oliva-Olivera W, Zayed H, Vega-Rioja A, Monteseirin J, Romero-Zerbo SY, Tinahones FJ, Bermudez-Silva FJ, El Bekay R, 2016. RPL13A and EEF1A1 are suitable reference genes for qPCR during adipocyte differentiation of vascular stromal cells from patients with different BMI and HOMA-IR. PLoS One 11, e0157002 doi:10.1371/journal.pone.0157002. 27304673 PMC4909211

[R14] Ghio AJ, Devlin RB, 2001. Inflammatory lung injury after bronchial instillation of air pollution particles. Am.J. Respir. Crit. Care Med 164, 704–708. doi:10.1164/ajrccm.164.4.2011089. 11520740

[R15] He Q, Guo W, Zhang G, Yan Y, Chen L, 2015. Characteristics and seasonal variations of carbonaceous species in PM2.5 in Taiyuan, China. Atmosphere 6, 850–862. doi:10.3390/atmos6060850.

[R16] Huang R-J, Cao J, Chen Y, Yang L, Shen J, You Q, Wang K, Lin C, Xu W, Gao B, Li Y, Chen Q, Hoffmann T, Dowd CD, Bilde M, Glasius M, 2018. Organosulfates in atmospheric aerosol: synthesis and quantitative analysis of PM2.5 from Xi’an, northwestern China. Atmos. Meas. Tech 11, 3447–3456. doi: 10.5194/amt-11-3447-2018.

[R17] Kelly FJ, Fussell JC, 2012. Size, source and chemical composition as determinants of toxicity attributable to ambient particulate matter. Atmos. Environ 60, 504–526. doi:10.1016/j.atmosenv.2012.06.039.

[R18] Kim KH, Kabir E, Kabir S, 2015. A review on the human health impact of airborne particulate matter. Environ. Int 74, 136–143. doi:10.1016/j.envint.2014.10.005. 25454230

[R19] Krewski D, Burnett R, Jerrett M, Pope CA, Rainham D, Calle E, Thurston G, Thun M, 2005. Mortality and long-term exposure to ambient air pollution: ongoing analyses based on the American Cancer Society cohort. J. Toxicol. Environ. Health A 68, 1093–1109. doi:10.1080/15287390590935941. 16024490

[R20] Lee BJ, Kim B, Lee K, 2014. Air pollution exposure and cardiovascular disease. Toxicol. Res 30, 71–75. doi:10.5487/TR.2014.30.2.071. 25071915 PMC4112067

[R21] Li YJ, Takizawa H, Azuma A, Kohyama T, Yamauchi Y, Kawada T, Kudoh S, Sugawara I, 2009. The effects of oxidative stress induced by prolonged low-dose diesel exhaust particle exposure on the generation of allergic airway inflammation differ between BALB/c and C57BL/6 mice. Immunopharmacol. Immunotoxicol 31, 230–237. doi:10.1080/08923970802383316. 18791914

[R22] Li YJ, Sun Y, Zhang Q, Li X, Li M, Zhou Z, Chan CK, 2017. Real-time chemical characterization of atmospheric particulate matter in China: a review. Atmos. Environ 158, 270–304. doi:10.1016/j.atmosenv.2017.02.027.

[R23] Liu X, Zhang Y, Cheng S-H, Xing J, Zhang Q, Streets DG, Jang C, Wang W-X, Hao J-M, 2010. Understanding of regional air pollution over China using CMAQ, part I performance evaluation and seasonal variation. Atmos. Environ 44, 2415–2426. doi:10.1016/j.atmosenv.2010.03.035.

[R24] Liu Y, Sun J, Gou Y, Sun X, Li X, Yuan Z, Kong L, Xue F, 2018. A Multicity analysis of the short-term effects of air pollution on the chronic obstructive pulmonary disease hospital admissions in Shandong, China. Int.J. Environ. Res. Public Health 15, 15. doi:10.3390/ijerph15040774. PMC5923816 29673181

[R25] Ma M, Li S, Jin H, Zhang Y, Xu J, Chen D, Kuimin C, Yuan Z, Xiao C, 2015. Characteristics and oxidative stress on rats and traffic policemen of ambient fine particulate matter from Shenyang. Sci. Total Environ 526, 110–115. doi:10.1016/j.scitotenv.2015.04.075. 25918898

[R26] Maueroder C, Kienhofer D, Hahn J, Schauer C, Manger B, Schett G, Herrmann M, Hoffmann MH, 2015. How neutrophil extracellular traps orchestrate the local immune response in gout. J. Mol. Med. (Berl.) 93, 727–734. doi:10.1007/s00109-015-1295-x. 26002146

[R27] Narayanan S, Glasser A, Hu YS, McDermott AM, 2005. The effect of interleukin-1 on cytokine gene expression by human corneal epithelial cells. Exp. Eye Res 80, 175–183. doi:10.1016/j.exer.2004.08.027. 15670796

[R28] Noel A, Xiao R, Perveen Z, Zaman HM, Rouse RL, Paulsen DB, Penn AL, 2016. Incomplete lung recovery following sub-acute inhalation of combustion-derived ultrafine particles in mice. Part. Fibre Toxicol 13 doi:10.1186/s12989-016-0122-z. PMC4766714 26911867

[R29] Pampel J, 2017. Housekeeping Genes.. https://www.genomics-online.com/resources/16/5049/housekeeping-genes/.

[R30] Pardo M, Porat Z, Rudich A, Schauer JJ, Rudich Y, 2016. Repeated exposures to roadside particulate matter extracts suppresses pulmonary defense mechanisms, resulting in lipid and protein oxidative damage. Environ. Pollut 210, 227–237. doi:10.1016/j.envpol.2015.12.009. 26735168

[R31] Phalen RF, 2004. The particulate air pollution controversy. Nonlinearity Biol. Toxicol. Med 2 doi:10.1080/15401420490900245. PMC2659607 19330148

[R32] Plummer LE, Carosino CM, Bein KJ, Zhao Y, Willits N, Smiley-Jewell S, Wexler AS, Pinkerton KE, 2015. Pulmonary inflammatory effects of source-oriented particulate matter from California’s San Joaquin Valley. Atmos. Environ 119 (1994), 174–181. doi:10.1016/j.atmosenv.2015.08.043. PMC4639935 26568698

[R33] Rajak R, Chattopadhyay A, 2019. Short and long-term exposure to ambient air pollution and impact on health in India: a systematic review. Int. J. Environ. Health Res 1–25. doi:10.1080/09603123.2019.1612042. 31070475

[R34] Rohr AC, Wyzga RE, 2012. Attributing health effects to individual particulate matter constituents. Atmos. Environ 62, 130–152. doi:10.1016/j.atmosenv.2012.07.036.

[R35] Rutgers M, van Pelt MJ, Dhert WJ, Creemers LB, Saris DB, 2010. Evaluation of histological scoring systems for tissue-engineered, repaired and osteoarthritic cartilage. Osteoarthr. Cartil 18, 12–23. doi:10.1016/j.joca.2009.08.009. 19747584

[R36] Shen G, Wang W, Yang Y, Zhu C, Min Y, Xue M, Ding J, Li W, Wang B, Shen H, Wang R, Wang X, Tao S, 2010. Emission factors and particulate matter size distribution of polycyclic aromatic hydrocarbons from residential coal combustions in rural Northern China. Atmos. Environ 44, 5237–5243. doi: 10.1016/j.atmosenv.2010.08.042. PMC3811148 24179437

[R37] Silva RM, Teesy C, Franzi L, Weir A, Westerhoff P, Evans JE, Pinkerton KE, 2013. Biological response to nano-scale titanium dioxide (TiO2): role of particle dose, shape, and retention. J. Toxicol. Environ. Health A 76, 953–972. doi:10.1080/15287394.2013.826567. 24156719 PMC4370163

[R38] Smith KR, Veranth JM, Kodavanti UP, Aust AE, Pinkerton KE, 2006. Acute pulmonary and systemic effects of inhaled coal fly ash in rats: comparison to ambient environmental particles. Toxicol. Sci 93, 390–399. doi:10.1093/toxsci/kfl062. 16840564

[R39] Stanway D, 2017. China’s Top Coal Province Shanxi to Start Winter Pollution War Early. Reuters.

[R40] Sun X, Wei H, Young DE, Bein KJ, Smiley-Jewell SM, Zhang Q, Fulgar CCB, Castaneda AR, Pham AK, Li W, Pinkerton KE, 2017. Differential pulmonary effects of wintertime California and China particulate matter in healthy young mice. Toxicol. Lett 278, 1–8. doi:10.1016/j.toxlet.2017.07.853. 28698096 PMC5572813

[R41] Untergasser A, Cutcutache I, Koressaar T, Ye J, Faircloth BC, Remm M, Rozen SG, 2012. Primer3-new capabilities and interfaces. Nucleic Acids Res. 40, e115 doi:10.1093/nar/gks596. 22730293 PMC3424584

[R42] Wang YC, Huang RJ, Ni HY, Chen Y, Wang QY, Li GH, Tie XX, Shen ZX, Huang Y, Liu SX, Dong WM, Xue P, Fröhlich R, Canonaco F, Elser M, Daellenbach KR, Bozzetti C, El Haddad I, Prévôt ASH, Canagaratna MR, Worsnop DR, Cao JJ, 2017. Chemical composition, sources and secondary processes of aerosols in Baoji city of northwest China. Atmos. Environ 158, 128–137. doi:10.1016/j.atmosenv.2017.03.026.

[R43] WHO, 2018. Ambient (outdoor) Air Quality and Health. World Health Organization. https://www.who.int/news-room/fact-sheets/detail/ambient-(outdoor)-air-quality-and-health.

[R44] WHO, 2019. How Air Pollution Is Destroying Our Health. . https://www.who.int/airpollution/news-and-events/how-air-pollution-is-destroying-our-health.

[R45] Wyzga RE, Rohr AC, 2015. Long-term particulate matter exposure: attributing health effects to individual PM components. J. Air Waste Manag. Assoc 65, 523–543. doi:10.1080/10962247.2015.1020396. 25947312

[R46] Xia Z, Duan X, Tao S, Qiu W, Liu D, Wang Y, Wei S, Wang B, Jiang Q, Lu B, Song Y, Hu X, 2013. Pollution level, inhalation exposure and lung cancer risk of ambient atmospheric polycyclic aromatic hydrocarbons (PAHs) in Taiyuan, China. Environ. Pollut 173, 150–156. doi:10.1016/j.envpol.2012.10.009. 23202645

[R47] Yang X, Teng F, 2018. The air quality co-benefit of coal control strategy in China. Resour. Conserv. Recycl 129, 373–382. doi:10.1016/j.resconrec.2016.08.011.

[R48] Yang L, Zhou X, Wang Z, Zhou Y, Cheng S, Xu P, Gao X, Nie W, Wang X, Wang W, 2012. Airborne fine particulate pollution in Jinan, China: concentrations, chemical compositions and influence on visibility impairment. Atmos. Environ 55, 506–514. doi:10.1016/j.atmosenv.2012.02.029.

[R49] Yuan W, Fulgar CC, Sun X, Vogel CFA, Wu CW, Zhang Q, Bein KJ, Young DE, Li W, Wei H, Pinkerton KE, 2020. In vivo and in vitro inflammatory responses to fine particulate matter (PM_2.5_) from China and California. Toxicol. Lett 328, 9. doi:10.1016/j.toxlet.2020.04.010. PMC7641014 32320776

[R50] Zhang J, Fulgar CC, Mar T, Young DE, Zhang Q, Bein KJ, Cui L, Castaneda A, Vogel CFA, Sun X, Li W, Smiley-Jewell S, Zhang Z, Pinkerton KE, 2018. TH17-induced neutrophils enhance the pulmonary allergic response following BALB/c exposure to house dust mite allergen and fine particulate matter from California and China. Toxicol. Sci 164, 627–643. doi:10.1093/toxsci/kfy127. 29846732 PMC6061684

