# Supplementary material for: Pulmonary health effects of wintertime particulate matter from California and China following repeated exposure and cessation
Source: Toxicol Lett. Author manuscript; Available in PMC 2022 Jan 1. (PMC8671358; doi:10.1016/j.toxlet.2021.10.014)
Supplement: supplement [file NIHMS1756455-supplement-supplement.doc]

Supplementary Table S1. Semi-Quantitative histopathology scoring rubric

| **Type of Pathology** | **Score** | | | |
| --- | --- | --- | --- | --- |
|  | **0** | **1** | **2** | **3** |
| **Alveolitis** | Normal. Thin alveolar walls, with very few free macrophages in the lumen. | Mild inflammation. Similar to 0 score with more free macrophages in the alveolar lumen. | Moderate inflammation, with the majority of the alveolar spaces.  Inflammatory cell types include macrophages, monocytes. | Marked influx of macrophages into the alveolar lumen forming large cellular agglomerates which occupy much of the airspace. |
| **Bronchiolitis** | Normal respiratory epithelium, no inflammatory cells present around the bronchiole and adjacent perivascular region(s). | Mild influx of macrophages and/or monocytes to the airway submucosa, but no neutrophils. | Slightly thickened airway due to moderate influx of macrophages into the submucosa. | Marked influx of macrophages and some neutrophils into the submucosal layer causing thickening of the airway. |
|  |  |  |  |  |

Supplementary Table S2. Mouse primers used for quantitative real-time polymerase chain reaction.

| Gene | Forward | Reverse |
| --- | --- | --- |
| *EEF1A1* | 5’-GCATGGTGGTTACCTTTGCT-3’ | 5’-CAGCAACATTGCCTCGTCTA-3’ |
| *IL-1β* | 5’-GGGCCTCAAAGGAAAGAATC-3’ | 5’-TACCAGTTGGGGAACTCTGC-3’ |
| *TNF-α* | 5’-AGCCCCCAGTCTGTATCCTT-3 | 5’-CTCCCTTTGCAGAACTCAGG-3’ |
| *CXCL-3* | 5’-CAACGGTGTCTGGATGTGTC-3’ | 5’-AGCCAAGGAATACTGCCTCA-3’ |
| *CXCL-5* | 5’-GAAAGCTAAGCGGAATGCAC-3’ | 5’-GGGACAATGGTTTCCCTTTT-3’ |
